# Language Bias in Health Research: External Factors That Influence Latent Language Patterns

**DOI:** 10.3389/frma.2020.00004

**Published:** 2020-08-20

**Authors:** Danny Valdez, Patricia Goodson

**Affiliations:** ^1^Department of Applied Health Science, Indiana University School of Public Health, Bloomington, IN, United States; ^2^Department of Health and Kinesiology, Texas A&M University, College Station, TX, United States

**Keywords:** topic models, language framing, ethics, reviews, publication history

## Abstract

**Background:** Concerns with problematic research are primarily attributed to statistics and methods used to support data. Language, as an extended component of problematic research in published work, is rarely given the same attention despite language's equally important role in shaping the discussion and framings of presented data.

**Purpose:** This study uses a topic modeling approach to study language as a predictor of potential bias among collected publication histories of several health research areas.

**Methods:** We applied Latent Dirichlet Allocation (LDA) topic models to dissect publication histories disaggregated by three factors commonly cited as language influencers: (1) time, to study ADHD pharmacotherapy; (2) funding source, to study sugar consumption; and (3) nation of origin, to study Pediatric Highly-Active Anti-Retroviral Therapy (P-HAART).

**Results:** We found that, for each factor, there were notable differences in language among each corpus when disaggregated by each factor. For time, article content changed to reflect new trends and research practices for the commonly prescribed ADHD medication, Ritalin. For funding source, industry and federally funded studies had differing foci, despite testing the same hypothesis. For nation of origin, regulatory structures between the United States and Europe seemingly influenced the direction of research.

**Conclusion:** This work presents two contributions to ethics research: (1) language and language framing should be studied as carefully as numeric data among studies of rigor, reproducibility, and transparency; and (2) the scientific community should continue to apply topic models as mediums to answer hypothesis-driven research questions.

## Introduction

Peer-reviewed research is facing unprecedented retraction rates for published work (Fang et al., 2012), in part due to an ongoing replicability crisis, by which scientists cannot recreate findings of published studies even under identical study conditions (Pashler and Wagenmakers, 2012). In such cases, numeric data quality is commonly identified as the primary area of concern (Earp and Trafimow, 2015). That is, the inability to replicate findings is generally assumed to be the fault of study data itself or the methods used to analyze it. The language employed to communicate those data is often ignored. However, failing to assess a researcher's language choices in tandem with their reporting practices unduly ignores an important piece of the puzzle—that the language used in scientific reporting can misrepresent research findings in a manner analogous to falsifying or incorrectly analyzing numeric data.

Although language-bias studies are common in media and linguistics fields, they are less common in the applied sciences, where success is often measured by Kreiman and Maunsell (2011). Consequently, the scope of this issue—i.e., how language can misrepresent data—remains understudied. However, given that a scientific article is published every 20 seconds and retractions due to false claims, or accidental mistakes, have increased by 300% among leading publishing groups (Marcus and Oransky, 2014), biased language and language framing represent a growing concern affecting the merit of science that should be studied more intently.

The purpose of this study is to explore language framing and its effect on the presentation of scientific findings. This paper intends to frame language as an equally important contributor to problematic science by answering the following question—to what extent do various factors, including time, funding source, and a study's nation of origin, influence latent language patterns in published research? To answer this question, we employ a topic-modeling approach to conventional content analyses. This family of techniques, developed in computer informatics, is designed to detect underlying latent structures in large amounts of data, including the language patterns in text, and the influence of various factors on text data. Within this paper we aim to apply these topic modeling frameworks to (1) identify and discuss how language is easily influenced by external factors; and (2) demonstrate how tools such as topic models can detect language variability in a less subjective manner by analyzing the publication histories of various health-related fields. Together, we hope to promote dialogues in the academia that emphasize language's role in shaping or framing scientific discussions. Importantly, we intend to validate this area of study—language framing—as equally important to investigations of bias.

### Language and Framing

Readers of all published materials, generally, hold an implicit assumption that the communication is objective and written in clear, unequivocal terms. In practice, however, the manner in which language is written and contextualized (e.g., rhetorical strategies, surreptitious wording, and withholding of details) may bias how the message is understood. This practice is known as framing, which, in communication literature, generally refers to how messages are strategically crafted to convey a message in a specific way (Chong and Druckman, 2007). Though the academic reporting genre aims to demonstrate the use of rigorous and objective science through “reliable and significant” findings, academic papers are not immune to inappropriate uses of framing devices (Harmon and Gross, 2007). Indeed, in any field, there is a risk that some may frame language to inappropriately bolster the merit of work, even if the language is untruthful. In some cases, this may result in a publication based on false or misleading language that would have otherwise been rejected if more factual language had been used. In a notorious example, Brian Wansink—a nutritional psychologist formerly at Cornell University—was accused of misrepresenting study findings through data fabrication (numeric) *and* sensationalizing findings (linguistic) to create mainstream appeal of his science (Dahlberg, 2018). While data fabrication and incorrect analyses were the primary accusations lobbied against Wansink (which among other practices included data-dredging and p-hacking), it was the framing used to sell appeal to media outlets (e.g., *Jesus Christ Supersize? The Growing Last Supper*) that led to increased scrutiny of his data and methods employed to generate his findings.

Surreptitious use of language and framing represents serious ethical misconduct, as it violates the implicit contract between authors and readers, operating in good faith, to provide factual, objective, and bias-free reporting of findings. As stated previously, however, studies of research bias commonly focus on numeric data and overlook linguistic devices used to frame problematic data. This may stem from the lack of systematic approaches to objectively evaluate the truthfulness/merit of linguistic framing. Unlike numeric bias—which has objective tools for its detection and measurement (i.e., meta-analyses and open-science initiatives that require submitting raw data for review and publication (Barden et al., 2006; McArdle, 2011)—language bias has no such measures. Indeed, identifying biased text remains a largely subjective enterprise when compared to available tools and resources for evaluating the merit and validity of quantitative outcomes (Drapeau, 2002). Further complicating the matter, without such measures, accusations of bias in research findings can, in turn, lead to accusations of bias against the accuser—i.e., attacking the researcher over evaluating the science. However, the limited means to evaluate linguistic framing in published studies does not diminish the importance of studying linguistic framing. Indeed, even the most poorly collected, sloppy data are likely to find a publication outlet if the manuscript is strongly written (Thompson, 1995). Therefore, while problematic data are often identified as the primary source of the replication crisis (Peng, 2015), we are only exploring half of the problem if we continue to ignore the equal role language plays in presenting and supporting findings.

### Topic Modeling

Topic modeling is a computer informatics tool used to mine large collections of text (known as a corpus) to identify commonly occurring themes across documents (Wang et al., 2018). The theoretical logic of topic modeling assumes that, in any corpus, there are latent thematic structures; however, these underlying themes are often undetectable given the sheer volume of “noise” embedded in the text content (Underwood, 2012). Therefore, we apply topic models to consolidate text and reduce linguistic noise to reveal only the most salient themes—or, the main ideas of the corpus. While there are many different forms of topic modeling (Latent Semantic Analysis [LSA], Topic Evolution Model [referred to as CTM], among others), the most widely used is Latent Dirichlet Allocation or LDA (Blei et al., 2003; Hoffman et al., 2010).

LDA uses Bayesian inferencing and Gibbs sampling to compare each word (x) with all other words (y) across the entire corpus to identify which words, and groups of words, are most probabilistically associated with one another (Griffiths and Steyvers, 2004; Steyvers and Griffiths, 2007; Porteous et al., 2008). Words with high probabilities of association are grouped together to form a cluster (or theme) while words that provide no structural meaning (i.e., prepositions and articles) are systematically eliminated from the corpus (Wang et al., 2018). Ideally, the words in each theme are similar enough that interpreting the thematic meaning of the grouped words is intuitive. For example, Barry et al. (2018) used LDA to examine the advertising practices of leading alcohol brands through archived social media feeds. They found that specific brands paired their products to language that matched respective marketing strategies (e.g., Malibu rum, a coconut-flavored liquor, was associated with *summer, beach, sun, coconut)*.

Valdez et al. (2018) have called for the adoption of topic modeling as a legitimate methodological tool in social and applied science fields such as health promotion. More important is those authors' contention that the scope of topic modeling analyses—which are primarily exploratory—should be used to answer more sophisticated and applied research questions:

While not exhaustive, here we propose three social sciences domains in which researchers could employ and expand the use of topic modeling: (1) as a tool for reducing unintentional reviewer bias in systematic literature reviewing, (2) for practical thematic exploration of qualitative data and thematic analysis validation, and (3) for comparing similar corpora to explore semantic similarities and differences. (2018, p. 11).

Indeed, current exploratory topic modeling applications only seek to consolidate large collections of text and identify overall themes. The analytic capabilities of topic modeling, however, extend beyond this exploratory lens. With regard to the subjectivity of challenging linguistic framing, approaches that include topic models to consolidate and map themes among text could provide a more objective framework with which to detect linguistic biases. Specifically, by using a machine to consolidate and thematically map scientific literature—i.e., archives of published research—one could compare these corpora for semantic differences when disaggregated by factors that are historically known for introducing bias into the science. This could, in turn, uncover important nuances across corpora that may demonstrate how these factors may intentionally or unintentionally be influencing language patterns.

### Language-Influencing Factor

A language-altering (or influencing) factor is defined here as any decision, action, or contribution to the research process that carries the potential to influence specific word choices (McArdle, 2011). Though there are numerous potential language-altering factors (see Delgado-Rodríguez and Llorca, 2004), here we explore three that are known to influence study findings: (1) time, (2) funding source, and (3) nation of origin. We selected these three factors because they represent tangible avenues by which semantic language differences may be most observable. First, advances in research and technical innovations are certainly reflected in scientific reports over time (e.g., HIV-related discourse evolving from terminal illness to chronic infection). Chronicling that change by examining the evolution of a specific area of study could identify historical points that spurred change or an innovation. Second, we selected funding source as vested interests represent one of the most visible sources of contamination in research and language patterns (e.g., federal vs. industry funding) (Chopra, 2003; Barden et al., 2006). Comparing corpora disaggregated by funding source may identify important nuances between funding mechanisms worthy of additional discussion. Finally, we selected nation of origin because of regulatory differences governing research across the globe (Van Norman, 2016). Those regulations may promote differing opinions and practices in a given field, which may manifest in the language used to relay scientific findings (Arrow and Aronson, 2016).

## Methods

This study is a machine-learning-based content analysis of abstracts from published studies collected via online repositories, including PubMed, EbscoHost, and Web of Science.

**Figure 1:**
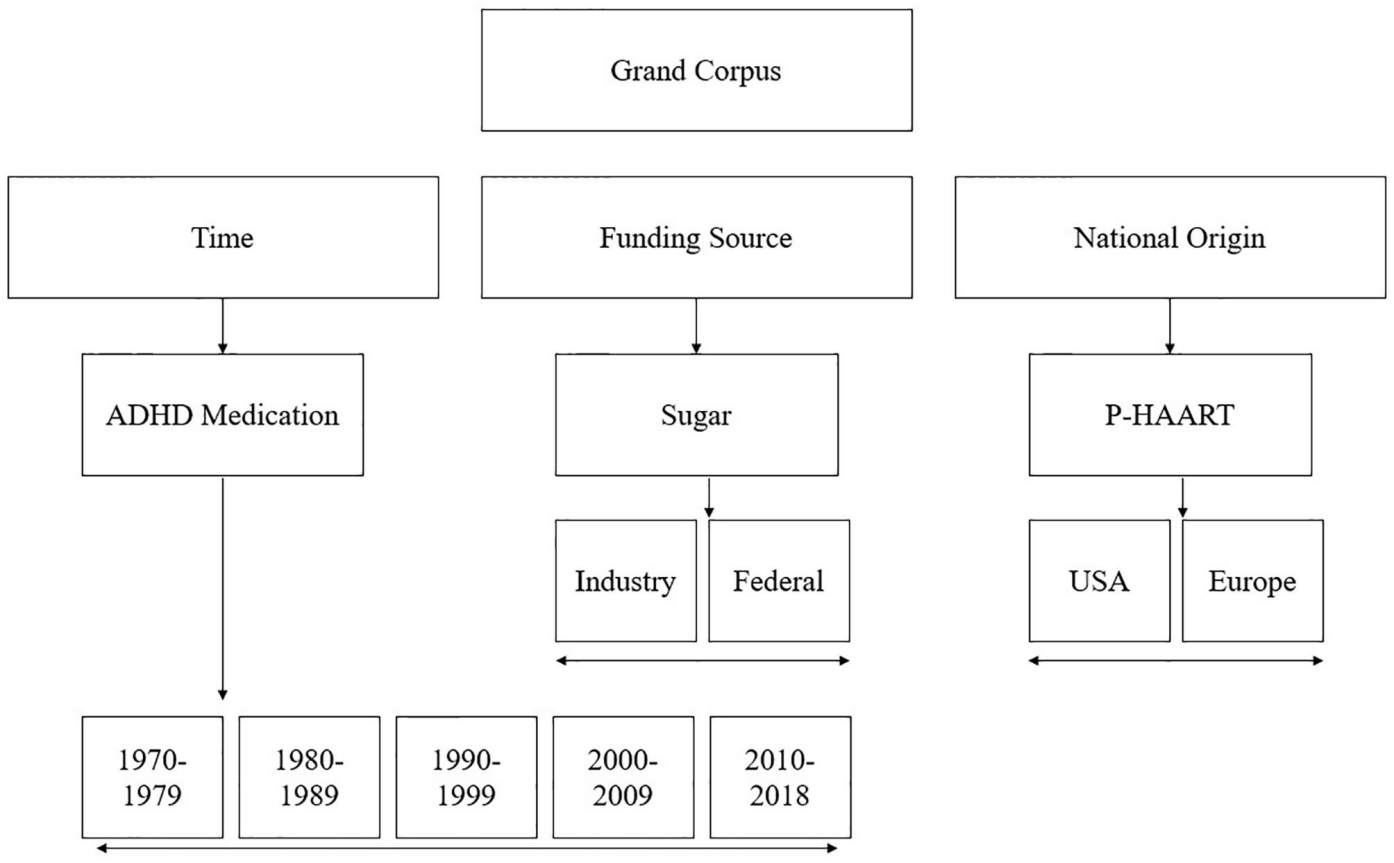
A diagram denoting corpora breakdown.

Our aim is to highlight how language used to frame these scientific reports may change when they are disaggregated, and compared, by various factors: time, funding source, and nation of origin. Our intent with this paper is not to scrutinize one, or any, area of research but, rather, to call attention to linguistic framing broadly. Therefore, to compare corpora by time, funding source, and nation of origin we purposefully collected three groups of abstracts from mutually exclusive research areas: ADHD pharmacotherapy (to test language changes over time), sugar consumption (to test language differences by funding source), and pediatric Highly Active Anti-retroviral Therapy (P-HAART) (to assess language differences between the United States and European Union). See Figure 1 for corpora breakdown.

We selected these specific content areas for several reasons. First, each of these areas (i.e., ADHD pharmacotherapy, sugar consumption, and P-HAART) is a prolific area of study, meaning there is a high volume of scientific output per each area. As such, these fields provide a rich library of language data to generate clear topic models for each language-altering factor (Hoffman et al., 2010). Second, these areas of study have also faced high rates of scrutiny among the scientific community and general public: ADHD is considered an over diagnosed condition, but ADHD pharmacotherapy represents a highly profitable drug market (Bruchmüller et al., 2012); objective sugar research is viewed as contaminated by industry sources (Kearns et al., 2016); and many P-HAART guidelines are considered to be lagging behind innovative scientific advances that require less invasive medication protocols (Mirani et al., 2015); and while these three fields are not representative of *all* health research, they still embody distinct heuristic examples with which to catch differences in linguistic framing within this small-scale study.

### Corpora

#### Time

This corpus, composed of ADHD pharmacotherapy research, sought to test if linguistic framing would gradually change over time to reflect advances in ADHD treatment. To build this corpus, we searched Pubmed, EbscoHost, Web of Science, and Medline using the most commonly prescribed ADHD medications as search terms: Ritalin, Concerta, Daytrana, RitalinLA, and Metadate. After removing duplicate entries, we retained 5,216 unique abstracts published from 1970 to 2018. The abstracts were subcategorized further into respective decades (i.e., 1970–1979, 1980–1989, … 2010–2018) for analysis. We generated one topic model for each 5 year increment, then compared those models, respectively.

#### Funding Source

This corpus, comprised of articles testing the link between sugar consumption and poor health-related outcomes, sought to evaluate linguistic framing in studies with different funding mechanisms. To compose this corpus, we searched PubMed, EbscoHost, Web of Science, and Medline, using various combinations of the terms “sugar” and “diet,” which, after excluding duplicates, yielded 828 unique abstracts. We then narrowed these abstracts further by removing articles that did not expressly indicate either federal funding (e.g., National Institutes of Health, Centers for Disease Control and Prevention, the Food and Drug administration, and others) or industry funding (e.g., PepsiCo, Coca Cola, Nestle Inc., and others) as the primary benefactor of the study. Our final abstracts included for analysis were 212 federally funded studies and 71 industry studies published from 2014 to 2018. We generated two topic models, one for federal and one for industry funding, and compared those models, respectively.

#### Nation of Origin

This corpus, composed of articles testing the efficacy of Pediatric HAART, sought to evaluate the linguistic framing of studies originating from either the United States or Europe. We intentionally selected this comparison to compliment the extended history of comparative EU/US medical research (Philipson, 2005; Lobo Abascal et al., 2016), in addition to the high rate at which studies in either region publish in the English language. To compose this corpus, we searched PubMed, EbscoHost, Web of Science, and Medline using various iterations of pediatric (or paediatric) HAART, including infant HAART and perinatal HAART. Our query returned 1,149 abstracts, excluding duplicates, which were evaluated further to determine if the study originated exclusively in the USA or a European country—including nation-specific samples and researchers. Because many studies included international research teams or were non-specific with their nation of origin, the corpus was significantly smaller: 74 US-based studies and 56 EU-based studies published between 2014 and 2018. We generated two topic models, one for US-based studies and one for EU-based studies, and compared those models, respectively.

### Analyses

All analyses, which included generating various LDA topic models for each language altering factor, were conducted using R version 3.4.2 and the following downloadable R packages: (1) topicmodels (sic), (2) tm, and (3) tidyR (sic). These packages—which are specialized program extensions for R—run text data through a multi-step process to prepare for analysis, including: (1) removing punctuation, numbers, special symbols (e.g., ^*^, <, >, &, among others), (2) stemming the document (i.e., removing all suffixes from words so that only the root word remains), and (3) creating a document term matrix, which is an aggregate calculation of how many times every word is used in a corpus, or sub-corpus.

Because topic models are an exploratory tool, there is little guidance regarding the appropriate number of topics and words per topic. Blei et al. (2003) note that, due to the lack of “fit” statistics in topic modeling methodologies, researchers should select the number of topic models that seem to accurately represent the data. In addition, Valdez et al. (2018) also highlight that, because the goal of topic models is to consolidate text, the structure of topic models, including the number of topics and words per topic, should be simple and manageable. As such, all generated topic models retained a concise 5 × 10 structure that included the five most important topics with the top 10 associated words in each topic.

We then assessed inter-rater reliability, a check for overall consistency in interpretations of qualitative data, with a qualitative researcher (Armstrong et al., 2016). Final results are presented below without comment.

## Results

### Time

This analysis sought to assess whether language employed in the reporting of ADHD pharmacotherapy studies changed over time. Because we archived nearly 50 years' worth of data, we divided the corpus into decade-spanning sub-corpora to compare differences among decades (see Table 1).

**Table 1:** ADHD Pharmacotherapy topic models, 1970–2018.

	**Topic 1**	**Topic 2**	**Topic 3**	**Topic 4**	**Topic 5**		**Topic 1**	**Topic 2**	**Topic 3**	**Topic 4**	**Topic 5**
	**1970–1979**						**1980–1989**				
1	Children	**Methyl**	Attent	Perform	Group	1	Studi	**Methyl**	Measur	Drug	Attent
2	Hyperact	**Effect**	Behavior	Learn	Rate	2	Task	**Hyperact**	Behavior	Disord	Differ
3	Drug	**Behavior**	Medic	Normal	Motor	3	Test	**Children**	Respons	Treatment	Rate
4	Hyperkinet	**Find**	Measure	Ritalin	Found	4	Condit	**Effect**	Hour	Activ	Stimul
5	Improve	**Stimul**	Condit	Treat	Height	5	Assess	**Boy**	Cognit	Concentr	Pharmacolog
6	Arous	**Abstract**	Control	Differ	Subject	6	Ritalin	**Deficit**	Improv	Subject	Present
7	Respons	**Hyperact**	Problem	Compar	Affect	7	Administr	**Perform**	Process	Medic	Meal
8	Report	**Physiology**	Test	Neurolog	Present	8	Mgkg	**Dose**	Prolactin	Add	Time
9	Treatment	**Show**	Dose	Task	Case	9	Reaction	**Increas**	Interact	Growth	Effect
10	Weight	**Respond**	Age	Dextroamp	Cognit	10	Group	**Studi**	Control	Posit	Design
	**1990–1999**						**2000–2009**				
1	Parent	Diagnosi	Function	Effect	**Methyl**	1	Focus	**Methyl**	Mode	Attent	Fluoxetin
2	Abus	Academ	Three	Children	**Disord**	2	Pfc	**Adhd**	Extens	Patient	Conflict
3	Assess	Diagnos	Human	Drug	**Adhd**	3	Characterist	**Effect**	Afternoon	Dose	Secondari
4	Amplitude	Remain	Stimulus	Hyperact	**Attent**	4	Neuropsycholog	**Mph**	Error	Hyperact	Communic
5	Experiment	Latenc	Sensit	Deficit	**Behavior**	5	Distribut	**Children**	Noradrenerg	Improv	Neurotransmiss
6	Potenti	Addit	Consist	Ritalin	**Stimul**	6	Place	**Disord**	Randomis	Differ	Rodent
7	Appear	Edsub	Stimuli	Medic	**Respons**	7	Biolog	**Drug**	Sexual	Year	Selfreport
8	Attribute	Emiss	Therapeut	Patient	**Dose**	8	Toxic	**Increas**	Pathway	Assess	Bid
9	Comparison	Issu	Tomogra	Test	**Mgkg**	9	Locat	**Medic**	Therebi	Suggest	Blind
10	Deficit	Lower	Addict	Cocain	**Report**	10	Valu	**Stimul**	Antidepress	Includ	Continu
	**2010–2018**										
1	**Adhd**	Improve	Roi	Neurochem	Adult						
2	**Methyl**	Day	Aetiolog	Basic	Perform						
3	**Mph**	Psycho	Fertil	Genotox	Present						
4	**Effect**	Cas	Produc	Belief	Function						
5	**Disord**	Cocain	Snps	Site	Baselin						
6	**Patient**	Receptor	Subcotr	Cage	Task						
7	**Drug**	Male	Methyl	Dawley	Administer						
8	**Children**	Common	Fix	Frontostriat	Atomoxetin						
9	**Medic**	Investing	Fli	Abl	Particip						
10	**Increase**	Time	Pkc	Arrest	Receiv						

Bolded columns represent the computer-identified most salient topic in the corpus. Of note, words in the most salient topic across all decades remained fairly consistent: *methyl, disord, adhd, mph, effect, behavior, drug*, among others. The remaining four (i.e., non-bolded, less salient) topics in each decade changed gradually over time. In the 1970s and 1980s, *children* and *boy* were common terms, potentially reflecting the populations most frequently addressed in the studies. Beginning in the 1990s, however, terms reflecting ADHD pharmacotherapy among other populations began to emerge often enough to appear within other latent topics, such as *parent, human* (in place of child), *rodent* and, in the 2010–18 sub-corpus, *adult*. Other words, such as *abuse, toxic*, and *addict*, begin appearing in the 1990s and beyond but remained entirely absent from older models. Table 2, derived from the document term matrix, depicts the rankings of words by frequency and co-occurrence with other words (i.e., how often words are used in a sub-corpus).

**Table 2:** Word ranking by decade on methylphenidate research.

	**1970–1979**	**1980–1989**	**1990–1999**	**2000–2009**	**2010–2018**
Boy	79	14	37	173	233
Girl	-	581	373	426	606
Adult	-	275	71	32	21
Adolescent	-	430	97	51	33
Toxic	673	-	551	704	931
Side	220	-	26	169	169
Adverse	179	-	231	97	129
Abuse	-	-	-	3219	104

In the 1970s, for example, the 79th most used word, *boy*, reflected the only population being tested— *girl, adolescent*, and *adult* did not appear in that decade's sub-corpus at all. Subsequent decades saw diversification regarding who was tested, eventually including girls, adults, and adolescents. Beginning in the late 90s, and extending into the 2010–2018 decade, the terms adult and adolescent became more important (i.e., more frequent) than the original 1970s term “*boy*.” Further, words such as abuse, adverse, and side (as in “side-effect”) also gained importance and became much more visible over time.

### Funding Source

This analysis sought to determine if funding source (i.e., industry or federal funding) for studies testing the link between table sugar and health comorbidities influenced language patterns. As shown in Table 3, language in both topic models was notably different.

**Table 3:** Topic models for industry and federally-funded research reports on sugar in the human diet.

	**Industry**						**Federal**				
	**Topic 1**	**Topic 2**	**Topic 3**	**Topic 4**	**Topic 5**		**Topic 1**	**Topic 2**	**Topic 3**	**Topic 4**	**Topic 5**
1	Total	Fruit	Chang	Calori	**Intak**	1	**Diet**	Fructose	Genet	Resist	Family
2	Increase	Weight	Product	Effect	**Sugar**	2	**Food**	Beverag	Program	Signal	Individu
3	Fructose	Mean	Reduct	Calor	**Diet**	3	**Sugar**	Metabol	Nutrient	Term	Random
4	Reduc	Breakfast	Design	Lower	**Consum**	4	**Intak**	Diseas	Lower	Larg	Women
5	Eat	Women	Effect	Trial	**Food**	5	**Increas**	Obes	Polici	Home	Store
6	Obes	Blood	Baselin	Free	**Energy**	6	**Weight**	Mice	Regress	Link	Amount
7	Well	Contribut	Promot	Examin	**Beverag**	7	**High**	Effect	Cost	Bodi	Insulin
8	Cvd	School	Measure	Obes	**Consumpt**	8	**Consump**	Insulin	Ssb	Gain	Loss
9	Loss	Carbohydr	Breakfast	Respect	**Dietary**	9	**Energi**	Relat	Ses	Progress	Analyz
10	Grain	Juic	Either	Observ	**Pattern**	10	**Risk**	Liver	Analys	Tumor	Healthi

Within the federally funded topic model, the most important topic contained the words *diet, food, sugar, intake, increase, weight, high, consumption, energy*, and *risk*. Topics 2, 4, and 5 centered on outcomes related to sugar consumption (e.g., *metabolism, disease, insulin, effect, mice, liver, link, bod, tumor*, among others), and topic 3 centered on interventions and cost (e.g., program, nutrient, *polici, cost, ssb* [a frequently used acronym for sugar sweetened beverages], *ses* [socio-economic status], and *regress*).

The industry-funded topic model seemed to have a different emphasis altogether. The most salient theme, Topic 5, contained the following words: *intake, sugar, diet, cosum, food, energy, beverage, consumpt, dietary*, and *pattern*. Diet, as in food consumed daily, was a recurrent theme in the majority of the remaining topics, especially observable in topics 2, 3, and 4. In those topics, food related words such as *calori, effect, baseline, promote, breakfast, fruit, juice, eat*, among others, were also common. Topic 1 in the industry-funded topic model, was notably different from topics 2, 3, 4, and 5. Rather than emphasize diet— as in food consumption—Topic 1 uniquely discussed outcomes of sugar consumption such as increased adiposity and heart function (e.g., *total, increase, fructose, reduce, eat, obes, cvd* [cardiovascular disease]).

### Nation of Origin

This analysis sought to determine if P-HAART studies conducted, funded, and published in the United States and in other European nations would influence language patterns. As with the previous analyses, there were indications of language differences between domestic and international studies (see Table 4).

**Table 4:** Topic models for European and US-based studies of P-HAART.

	**Europe**						**United States**				
	**Topic 1**	**Topic 2**	**Topic 3**	**Topic 4**	**Topic 5**		**Topic 1**	**Topic 2**	**Topic 3**	**Topic 4**	**Topic 5**
1	Year	**Art**	Therapi	Guideline	HIV	1	**HIV**	Drug	Test	Antiretrovir	Prevent
2	Present	**Parent**	Unit	Health	Children	2	**Infect**	Medic	Aid	Prophylaxi	Research
3	Patient	**Manag**	Survey	Recommend	Infect	3	**Children**	Birth	Born	Expos	Compar
4	Country	**Status**	Pediatr	Provid	Paediatr	4	**Pediatr**	Issu	Differ	Receiv	Evalu
5	Report	**Diagnos**	Count	Migrant	Care	5	**Health**	Virus	Recommend	Increas	Adult
6	Start	**Drug**	Develop	Aid	Age	6	**Report**	Famili	High	Guidelin	Particip
7	Antenat	**Escmid**	Four	Adolesc	Women	7	**Care**	Mhps	Behavior	Regimen	Physician
8	Acquir	**Screen**	Time	Diseas	Clinic	8	**Youth**	Transmiss	Caregiv	Factor	Present
9	Active	**Europ**	Case	Live	Follow	9	**Infant**	Viral	Exposur	Cdc	Patient
10	Differ	**Hundr**	Childhood	Mortal	European	10	**Diseas**	Resist	Matern	Human	Assess

Language in US-based studies focused on prescribing and administering of P-HAART to infants upon birth. The most-salient theme for US-based studies contained the following words: *HIV, infect, children, pediatr, health, report, care, youth, infant*, and *disease*. Topics 2 and 3 in the US-based model focused on HIV transmission and pharmacotherapy applications: *drug, medic, birth, issue, viral, test, aid, born, recommend, high, dose, exposure, matern, among others*. Topics 4 and 5 used slightly different words to convey a focus on general recommendations and federal guidelines, such as *regimin, factor, cdc, human, research evalu, adult*, and *patient*.

Similarly, the European studies also focused on medication adherence. However, these studies focused more on management of HIV rather than HAART uptake. The most-important theme contained the following words: *art, parent, manage, status, diagnos, drug, screen, europ, hundred*. Topic 3 reflects guidelines using the words guideline*, health, recommend, provid, migrant, aid, adolescent*, and *disease*. Topic 1, on the other hand, addresses national reports of HIV infection: *year, present, patient, country*, and *report*. Topic 5 can further be interpreted as care for children living with HIV: *HIV, children, infect, paediatri, care, age, women, clinic, follow, European*.

## Discussion

For each of the three language altering factors, the resultant topic models uncovered various linguistic differences that may be partially explained by the factors discussed above. We note that it is not our intent to accuse authors of being linguistically biased, we simply aim to highlight how the factors outlined up above can, and often do, play a role in shaping the direction of linguistic patterns and framing of published research. Below, we situate our findings within the context of their respective literatures to explain many of the differences identified in the topic models.

### Time

As noted, we observed linguistic changes in ADHD pharmacotherapy language between 1970 and 2018. Part of those changes may be partially explained by the growth of ADHD pharmacotherapy as a publishable research field over time. In the 1970s—roughly the start of the ADHD pharmacotherapy era—we collected fewer than 100 scientific publications on Ritalin and other ADHD-related medications. In the 2010 decade we collected nearly 3,000 unique abstracts from diverse fields, including psychology, sociology, biology, epidemiology, and others.

Within that growth, we captured changes regarding the intended demographic for ADHD pharmacotherapy. Specifically, children to whom Ritalin was first administered in the early 1980s grew into adulthood in the 1990s and early 2000s (Schachter et al., 2001), redirecting focus on ADHD pharmacotherapy from childhood and adolescence into adulthood. The desire to increase the scope of Ritalin was equally reflected in the topic models—in later years, the terms *girl, adolescent*, and *adult* eventually emerge as components in emergent topics. While this drug was initially intended to primarily treat children (specifically, boys), the shift in demographics prompted new clinical trials to determine if Ritalin regimens were safe long term (i.e., into adulthood) (Cox et al., 2000).

Due to successful efficacy and safety testing among adolescent and adult populations, guidelines governing ADHD pharmacotherapies adapted to include a patient population that did not consist merely of children (Conrad and Potter, 2000). For example, in 2001, guidelines published in the *Journal of Pediatrics* noted the appropriate age for Ritalin use was no younger than 6 years of age and no older than 12. In an update to those guidelines (in 2011) there were two major changes to reflect updated positions on ADHD and ADHD pharmacotherapies: first, ADHD was reclassified from a psychological disorder to a chronic condition, and second, the appropriate ages to administer Ritalin were changed to include children as young as 4 and adults 18 and over (American Academy of Pediatrics, 2011, 2019).

With adults, adolescents, and children now using Ritalin, the amount of prescriptions written for ADHD pharmacotherapy doubled within 1 decade (Hamed et al., 2015); and due to the wide availability and administrations of Ritalin and other ADHD pharmacotherapies, researchers were further able to document new aspects of Ritalin that were previously unstudied—such as its negative side effects, and addiction. Beginning in the 1990s Ritalin—once considered a safe drug intended to treat hyperactivity in children—was now classified as a high-risk study drug linked to abuse (Babcock and Byrne, 2000; Morton and Stockton, 2000). More importantly, the topic model was able to capture this important nuance—the term *abuse* would first appear as a topic in the 1990s model. Regardless, ADHD pharmacotherapy represents a multibillion dollar industry with ADHD diagnoses representing the second most frequent long-term diagnosis in children (Bergey et al., 2018).

### Funding Source

The topic model for the federally funded research was mainly comprised of language that emphasized comorbidities associated with sugar consumption. For example, words such as *risk, weight, gain, tumor, insulin, metabol*, and *disease* can be interpreted as describing health-related issues associated with sugar consumption, including increased adiposity and metabolic related diseases (Rodearmel et al., 2007). Similar language is paralleled in federal guidelines about sugar and health, such as those from the CDC, that aim to decrease sugar consumption in children and adults (Park, 2014). Specifically, “Americans are eating and drinking too much added sugars, which can lead to problems such as weight gain, type-2 diabetes, and heart disease” (CDC, 2019).

Diagnostic language, or language that highlights health-related comorbidities of sugar consumption, is almost absent from the industry funded topic model. This model seems to place sugar within the context of a normal part of the human diet, to be enjoyed in moderation, over highlighting the chronic conditions and co-morbidities that were emphasized in the federally funded model. Importantly, language in each of the topics in the industry-funded research model tended to pair sugar with other household items and behaviors often billed as healthy—such as *fruit, juice, grain*, and *breakfast*. Gambrill (2012), who contends some industry-based investigations are inherently biased, notes diverting attention away from serious outcomes as “oversimplification [used to] dull critical thinking” and mask lingering controversies (p. 289). Wolfson (2017) further adds that oversimplification is common among many types of research funded in-house, in a bid to mitigate a bad reputation; and because industry is often viewed as one of the biggest contaminators of objective research, it seems intuitive to cast the differing foci as indications of lower rigor within the industry-funded group.

However, those accusations may also be misguided without carefully reviewing industry funded studies further. When the UK's Academy of Medical Royal Colleges argued 30 min of moderate exercise five times weekly was more powerful than any drug at preventing chronic disease, Malhotra et al. (2015) who disclosed receiving funding from the Atkins Scientific Advisory Board—counterargued, “you cannot outrun a bad diet…[to] reduce the risk of cardiovascular disease [and] type 2 diabetes” (p. 967). Of note, important words in the editorial including *diet, cvd, and diabetes*, are also paralleled in our industry model. Their editorial is one example of numerous others funded, at least partially, by an industry that, like their federal counterparts, remains critical of sugar. However, because industry studies maintain a poor reputation, observed differences between industry and federal topic models seemed to be rooted in accusations of lower rigor. However, through a more careful review, it became apparent that those differences may primarily be attributed to different foci of these studies and not necessarily differences in study quality. Thus, we maintain critical examinations of research are essential, as it is easy to accuse one group over another of bias, especially without a thorough review. More thorough content analyses are needed to evaluate the rigor (and framing) differences in industry vs. federally funded sugar studies.

### Nation of Origin

Regarding nation of origin, the US topic model was clear regarding the targeted population: infants and children (e.g., *birth, issue, virus, transmiss, infant, hiv, children*). More evident was the sense of urgency in administering P-HAART at the time of birth: *birth, issue antiretrovir, prophylaxi, receive*. In the EU model, however, the target population was not as clear, as there were more emergent groups throughout the corpus and final topic model: *provid, migrant, aid, adolesc, women, children*. Absent altogether from the European model was the word *infant* despite this corpus being composed of studies regarding pediatric HAART.

As mentioned previously, these distinctions most likely stem from the regulatory differences between the United States and other nations in the EU. These differences may lead to conflicting perspectives of pharmacotherapy, generally, and recommendations outlined in research. For example, the United States, where 70% of the population takes at least one prescribed medication daily (Mayo Clinic, 2013), remains steadfast in pharmacotherapy for treatable illnesses, particularly those that are transmittable. The National Institutes of Health AIDS Guidelines expressly state:

“The uses of anti-retroviral (ARV) in infants include: “one or more ARV drugs to a newborn [immediately] without confirmed HIV infection to reduce the risk of HIV acquisition HIV *in utero*, during the birthing process or during breastfeeding and who do not acquire HIV” (National Institutes of Health, 2017, pg. H-1, emphasis added, retrieved at https://aidsinfo.nih.gov/contentfiles/lvguidelines/PediatricGuidelines.pdf). Simply, any infant is to begin P-HAART even before diagnosis is confirmed.”

By contrast, in Europe, where <40% of the population takes a daily prescription medication (Eurostat, 2018), the Pediatric European Network for Treatment of AIDS (PENTA) guidelines (2015) emphasize treatment of *older children/adolescents* and not pre-diagnosed infants: “PENTA guidelines seek to optimize treatment for children…particularly during adolescence, [when] care may need to be individualized…[additional]consideration of ART initiation in all children aged 1–3 years [is needed] in order to minimize risks of disease progression or death” (Bamford et al., 2018, p. e5).

Given the competing emphases between the NIH and PENTA guidelines, it is perhaps not entirely surprising that the US-based studies focused on younger children/infants. The wider acceptance of pharmacotherapy in the US mirrors the prevailing view to “hit HIV hard and early” to prevent transmission across populations through anti-retrovirals (Ho, 1995, p. 450). In the EU, however, pharmaceutical research and medication distribution are regulated heavily by the government. This regulation, in part, seeks to de-incentivize profiteering by pharmaceutical companies, which is viewed as a common problem in the US (Eger and Mahlich, 2014). Indeed, almost all major medications are significantly cheaper in the EU (Danzon and Chao, 2000). Therefore, due to regulations in which profit incentives are removed, any tested medication in Europe will be more widely scrutinized, evaluated, and thoroughly tested before ever being approved for use among the general population (Eger and Mahlich, 2014). This greater skepticism may explain why a documented case of HIV is needed before beginning anti-retroviral treatment.

### Language Framing and Problematic Science

As outlined in this study, we sought to test if various factors (i.e., time, funding source, and nation of origin) influenced language patterns presented in published, peer-reviewed research. Our findings illustrate that, through topic modeling, we successfully identified linguistic differences by each language influencing factor, including shifts in the intended demographic populations of a research field, and foci of the studies. In particular, these differences clearly demonstrate how scientific language aligns itself with the larger narratives within which it is embedded, meaning that external factors inevitably influence the direction of a research field. Given that our findings underscore the vulnerability of language to such factors, we argue that language framing in scientific reporting should be an equally important consideration (along with numeric data) when evaluating the merit of scientific work. Even if, through this study, we could not objectively declare instances of bias within collections of abstracts, the linguistic differences identified for each factor warrant pause for concern and evidence that further evaluations are needed to rigorously examine science from a linguistics perspective.

Importantly, our findings also situate topic modeling as a valid, more-objective public health tool with which to evaluate language and text, whether that be from published peer-reviewed literature, industry-based promotional advertising, public policy documents, and/or social media and internet content. Though this approach is still a novel in some fields, including health, tools such as topic modeling have been widely used in other fields to identify nuance within language amongst large collections of text. Thus, health promotion, public health practitioners, and other types of research should leverage this useful tool to further advance studies of bias—both numeric and language based—and ultimately improve health and well-being and the integrity of science within our fields.

## Limitations

All studies are subject to limitations, inclusive of this investigation. First, we acknowledge that despite online databases such as PubMed and EbscoHost containing full-text access to many published articles included in our study, we intentionally only selected abstracts for review. This was strategically done for two reasons. First, topic models are a means to consolidate language into manageable themes. Abstracts of scientific papers represent, “information that is the most important for the reader and is often used as a proxy for the content of an article” (Ermakova et al., 2018; Atanassova et al., 2019). Therefore, abstracts are a logical choice for analysis, as topic models using full-text information would, more likely than not, result in very similar output using much more computing power than necessary. Second, because not every researcher may have access to full-text libraries, we selected abstracts, which are generally free to access, to encourage replication of this study and future studies that mine scientific bodies of literature. Thus, any detraction due to the use of abstracts in this study is minimal and does not impact the validity of findings.

## Conclusion

Because the lay public often cannot differentiate between good and bad quality science, the obligation falls on scientists to uphold the credibility of their scientific endeavors by being transparent with the outcomes of their work (Wallach et al., 2018). Hubbard (2015) cautions that while all scientists have an agenda, not all agendas are created equally, and certain agendas seek profit over progress; and as Gambrill (2012) contends, the key to making an informed choice in society is access to quality information that clearly conveys its message with clear objectivity, lest we make ill-informed decisions supported by science of questionable quality. Concerns over both data manipulation and biasing language are equally important, as both contribute to the ongoing replicability crisis across the sciences. Given the rapid rate at which scientific research is being published, we argue for the need to more critically assess findings in the literature both linguistically and numerically. Further, readers should be better equipped to identify biasing factors, including through the use of novel tools and methodologies such as the topic modeling approach used here to help identify potentially biased language and framing.

## Data Availability Statement

The raw data supporting the conclusions of this article will be made available by the authors, without undue reservation, to any qualified researcher.

## Author Contributions

DV was the primary author and facilitated all aspects of this study, including conception, data collection, data analysis, and presentation of findings as part of a doctoral dissertation. PG oversaw the logic and framing of this study, in addition to providing assistance with writing. All authors contributed to the article and approved the submitted version.

## Conflict of Interest

The authors declare that the research was conducted in the absence of any commercial or financial relationships that could be construed as a potential conflict of interest.
